# 9-{[4-(Dimethyl­amino)­benzyl]amino}-5-(4-hy­droxy-3,5-dimeth­oxy­phenyl)-5,5a,8a,9-tetra­hydro­furo[3′,4′:6,7]naphtho­[2,3-*d*][1,3]dioxol-6(8*H*)-one methanol monosolvate

**DOI:** 10.1107/S1600536811043054

**Published:** 2011-10-29

**Authors:** Hong Chen, Dan-Li Tian, Hong Chen, Shao-Yu Shi, Ting Ai

**Affiliations:** a Affiliated Hospital of the Medical College of the Chinese People’s Armed Police Forces, Tianjin 300162, People’s Republic of China; b Room of Pharmacognosy, Medical College of the Chinese People’s Armed Police Forces, Tianjin 300162, People’s Republic of China; c Tianjin Key Laboratory for Biomarkers of Occupational and Environmental Hazards, Tianjin 300162, People’s Republic of China

## Abstract

In the title compound, C_30_H_32_N_2_O_7_·CH_4_O, the tetra­hydro­furan ring and the six-membered ring fused to it both display envelope conformations, with the ring C atom opposite the carbonyl group and the adjacent bridgehead C atom as the flaps, respectively. In the crystal structure, inter­molecular O—H⋯O hydrogen bonds link all moieties into ribbons along [010]. Weak inter­molecular C—H⋯O inter­actions consolidate the crystal packing further.

## Related literature

For the crystal structures of related podophyllotoxin derivatives, see: Luo *et al.* (2011 ▶); Li *et al.* (2011 ▶).
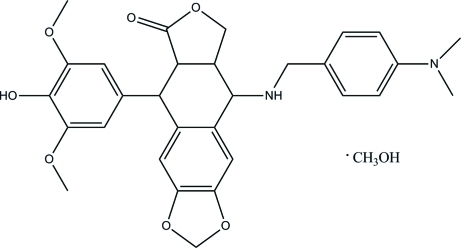


## Experimental

### 

#### Crystal data

C_30_H_32_N_2_O_7_·CH_4_O
*M* *_r_* = 564.62
Monoclinic, 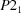

*a* = 11.128 (4) Å
*b* = 8.757 (3) Å
*c* = 15.057 (5) Å
β = 105.093 (6)°
*V* = 1416.7 (8) Å^3^
*Z* = 2
Mo *K*α radiation
μ = 0.10 mm^−1^
*T* = 113 K
0.20 × 0.18 × 0.12 mm

#### Data collection

Rigaku Saturn724 CCD diffractometer
Absorption correction: multi-scan (*CrystalClear*; Rigaku, 2007 ▶) *T*_min_ = 0.981, *T*_max_ = 0.989
14990 measured reflections
3602 independent reflections
3167 reflections with *I* > 2σ(*I*)
*R*_int_ = 0.060

#### Refinement

*R*[*F* ^2^ > 2σ(*F* ^2^)] = 0.052
*wR*(*F* ^2^) = 0.107
*S* = 1.06
3602 reflections
377 parameters
1 restraint
H-atom parameters constrained
Δρ_max_ = 0.33 e Å^−3^
Δρ_min_ = −0.36 e Å^−3^

### 

Data collection: *CrystalClear* (Rigaku, 2007 ▶); cell refinement: *CrystalClear*; data reduction: *CrystalClear*; program(s) used to solve structure: *SHELXS97* (Sheldrick, 2008 ▶); program(s) used to refine structure: *SHELXL97* (Sheldrick, 2008 ▶); molecular graphics: *SHELXTL* (Sheldrick, 2008 ▶); software used to prepare material for publication: *SHELXL97*.

## Supplementary Material

Crystal structure: contains datablock(s) I, global. DOI: 10.1107/S1600536811043054/cv5160sup1.cif

Structure factors: contains datablock(s) I. DOI: 10.1107/S1600536811043054/cv5160Isup2.hkl

Supplementary material file. DOI: 10.1107/S1600536811043054/cv5160Isup3.cml

Additional supplementary materials:  crystallographic information; 3D view; checkCIF report

## Figures and Tables

**Table 1:** Hydrogen-bond geometry (Å, °)

*D*—H⋯*A*	*D*—H	H⋯*A*	*D*⋯*A*	*D*—H⋯*A*
O8—H8*A*⋯O1	0.84	1.95	2.777 (3)	170
O6—H6*A*⋯O8^i^	0.84	1.97	2.756 (3)	156
C7—H7*B*⋯N1^ii^	0.99	2.62	3.473 (4)	145
C13—H13*B*⋯O6^iii^	0.99	2.52	3.463 (4)	159
C24—H24*A*⋯O3^iv^	0.95	2.35	3.242 (4)	156

Symmetry codes: (i) 
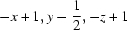
; (ii) 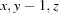; (iii) 
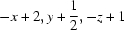
; (iv) 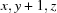.

**Fig. 1.**
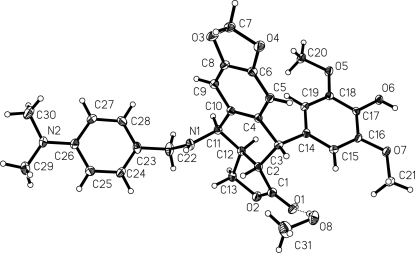
View of the title compound, with displacement ellipsoids drawn at the 40% probability level. Dashed line denotes hydrogen bond.

## supplementary crystallographic information

### Comment

In continuation of our structural study of new derivatives of Podophyllotoxin (Luo *et al.*, 2011; Li *et al.*, 2011), we report here the crystal structure of the title compound (I).

In (I) (Fig. 1), all bond lengths and angles are normal and in a good agreement with those reported previously for related compounds (Luo *et al.*, 2011; Li *et al.*, 2011). The tetrahydrofuran ring (C1/C2/C12/C13/O2) and the six-membered ring (C2–C4/C10–C12) fused to it both display envelope conformations. The dihedral angles between the benzene ring (C4–C6/C8–C10) of the benzo[*d*]-[1,3]dioxole and the other two benzene ring (C14–C19 and C23–C28) are 80.47 (3) and 71.32 (2)°, respectively.

In the crystal structure, intermolecular O—H···O hydrogen bonds (Table 1) link all moieties into ribbons in [010]. Weak intermolecular C—H···O interactions (Table 1) consolidate further the crystal packing.

### Experimental

The title compound was synthesized in two steps. A mixture of 4-dimethylamino carbaldehyde (0.179 g,1.2 mmol), 4β-amino-4-De methyl-epipodophyllotoxin (0.399 g,1 mmol) and two drops of acetic in 95% ethanol (30 ml) was stirred for 6 h, then an appropriate amount of NaBH_4_ was added and the mixture stirred for 2 h at 273 K. After the addition of 5% HCl (5 ml) to end off the reaction, the mixture was concentrated *in vacuo* and the pH adjusted to basic conditions with a saturated NaHCO_3_ solution. The reaction mixture was extracted with CH_2_Cl_2_, dried over MgSO_4_ and concentrated *in vacuo*. The residue was dissolved in methanol and slow evaporation over two weeks at room temperature gave colourless crystal suitable for X-ray analysis.

### Refinement

All H atoms were found on difference maps, but placed in idealized positions with C—H = 0.95–1.00 Å, N—H = 0.88 Å, O—H = 0.84 Å, and included in the final cycles of refinement using a riding model, with *U*_iso_(H) = 1.2-1.5*U*_eq_ of the parent atom. In the absence of significant anomalous scatterers in the molecule, 2757 Friedel pairs were merged before the final refinement.

### Crystal data

**Table:**

C_30_H_32_N_2_O_7_·CH_4_O	*F*(000) = 600
*M**_r_* = 564.62	*D*_x_ = 1.324 Mg m^−^^3^
Monoclinic, *P*2_1_	Mo *K*α radiation, λ = 0.71073 Å
Hall symbol: P 2yb	Cell parameters from 3904 reflections
*a* = 11.128 (4) Å	θ = 1.4–27.9°
*b* = 8.757 (3) Å	µ = 0.10 mm^−^^1^
*c* = 15.057 (5) Å	*T* = 113 K
β = 105.093 (6)°	Prism, colourless
*V* = 1416.7 (8) Å^3^	0.20 × 0.18 × 0.12 mm
*Z* = 2	

### Data collection

**Table:**

Rigaku Saturn724 CCD diffractometer	3602 independent reflections
Radiation source: rotating anode	3167 reflections with *I* > 2σ(*I*)
multilayer	*R*_int_ = 0.060
Detector resolution: 14.22 pixels mm^-1^	θ_max_ = 27.9°, θ_min_ = 1.4°
ω and φ scans	*h* = −14→14
Absorption correction: multi-scan (*CrystalClear*; Rigaku, 2007)	*k* = −11→11
*T*_min_ = 0.981, *T*_max_ = 0.989	*l* = −19→19
14990 measured reflections	

### Refinement

**Table:**

Refinement on *F*^2^	Primary atom site location: structure-invariant direct methods
Least-squares matrix: full	Secondary atom site location: difference Fourier map
*R*[*F*^2^ > 2σ(*F*^2^)] = 0.052	Hydrogen site location: inferred from neighbouring sites
*wR*(*F*^2^) = 0.107	H-atom parameters constrained
*S* = 1.06	*w* = 1/[σ^2^(*F*_o_^2^) + (0.0493*P*)^2^] where *P* = (*F*_o_^2^ + 2*F*_c_^2^)/3
3602 reflections	(Δ/σ)_max_ < 0.001
377 parameters	Δρ_max_ = 0.33 e Å^−^^3^
1 restraint	Δρ_min_ = −0.36 e Å^−^^3^

### Special details

**Table:**

Geometry. All e.s.d.'s (except the e.s.d. in the dihedral angle between two l.s. planes) are estimated using the full covariance matrix. The cell e.s.d.'s are taken into account individually in the estimation of e.s.d.'s in distances, angles and torsion angles; correlations between e.s.d.'s in cell parameters are only used when they are defined by crystal symmetry. An approximate (isotropic) treatment of cell e.s.d.'s is used for estimating e.s.d.'s involving l.s. planes.
Refinement. Refinement of *F*^2^ against ALL reflections. The weighted *R*-factor *wR* and goodness of fit *S* are based on *F*^2^, conventional *R*-factors *R* are based on *F*, with *F* set to zero for negative *F*^2^. The threshold expression of *F*^2^ > σ(*F*^2^) is used only for calculating *R*-factors(gt) *etc*. and is not relevant to the choice of reflections for refinement. *R*-factors based on *F*^2^ are statistically about twice as large as those based on *F*, and *R*-factors based on ALL data will be even larger.

### Fractional atomic coordinates and isotropic or equivalent isotropic displacement parameters (Å^2^)

**Table:**

	*x*	*y*	*z*	*U*_iso_*/*U*_eq_	
O1	0.74921 (18)	0.6561 (3)	0.65328 (15)	0.0284 (5)	
O2	0.95083 (18)	0.7007 (3)	0.66811 (13)	0.0252 (5)	
O3	1.1227 (2)	−0.1440 (3)	0.94087 (15)	0.0310 (5)	
O4	0.90857 (19)	−0.1737 (3)	0.89208 (14)	0.0286 (5)	
O5	0.89789 (18)	0.0075 (3)	0.47434 (13)	0.0238 (5)	
O6	0.67582 (17)	0.0835 (3)	0.37010 (12)	0.0228 (5)	
H6A	0.5991	0.0978	0.3484	0.034*	
O7	0.52790 (17)	0.2841 (3)	0.42785 (13)	0.0250 (5)	
N1	1.1718 (2)	0.4576 (3)	0.89519 (15)	0.0218 (6)	
H1A	1.1346	0.4563	0.9401	0.026*	
N2	1.6236 (3)	0.5827 (4)	1.2607 (2)	0.0444 (8)	
C1	0.8569 (3)	0.6166 (4)	0.68409 (19)	0.0221 (7)	
C2	0.9091 (2)	0.4829 (4)	0.74486 (18)	0.0189 (6)	
H2A	0.9185	0.5164	0.8098	0.023*	
C3	0.8398 (2)	0.3297 (3)	0.73304 (18)	0.0170 (6)	
H3A	0.7638	0.3427	0.7560	0.020*	
C4	0.9242 (2)	0.2135 (4)	0.79506 (17)	0.0167 (6)	
C5	0.8672 (3)	0.0786 (4)	0.81573 (18)	0.0197 (6)	
H5A	0.7797	0.0649	0.7948	0.024*	
C6	0.9408 (3)	−0.0313 (4)	0.86629 (18)	0.0201 (6)	
C7	1.0196 (3)	−0.2290 (4)	0.9563 (2)	0.0255 (7)	
H7A	1.0138	−0.2139	1.0202	0.031*	
H7B	1.0307	−0.3393	0.9465	0.031*	
C8	1.0694 (3)	−0.0135 (4)	0.89553 (19)	0.0226 (7)	
C9	1.1282 (3)	0.1132 (4)	0.8767 (2)	0.0223 (6)	
H9A	1.2162	0.1225	0.8971	0.027*	
C10	1.0542 (3)	0.2319 (3)	0.82540 (17)	0.0178 (6)	
C11	1.1241 (2)	0.3740 (4)	0.80762 (18)	0.0190 (6)	
H11A	1.1972	0.3398	0.7857	0.023*	
C12	1.0402 (2)	0.4690 (4)	0.73161 (19)	0.0194 (6)	
H12A	1.0337	0.4155	0.6718	0.023*	
C13	1.0703 (3)	0.6355 (4)	0.7195 (2)	0.0222 (7)	
H13A	1.1012	0.6865	0.7798	0.027*	
H13B	1.1337	0.6453	0.6843	0.027*	
C14	0.7977 (2)	0.2702 (4)	0.63358 (18)	0.0171 (6)	
C15	0.6814 (3)	0.3129 (3)	0.57749 (18)	0.0187 (6)	
H15A	0.6304	0.3826	0.5995	0.022*	
C16	0.6410 (2)	0.2524 (4)	0.48918 (18)	0.0186 (6)	
C17	0.7132 (2)	0.1481 (3)	0.45567 (17)	0.0170 (6)	
C18	0.8302 (2)	0.1073 (3)	0.51183 (18)	0.0172 (6)	
C19	0.8707 (2)	0.1677 (4)	0.60009 (18)	0.0190 (6)	
H19A	0.9496	0.1384	0.6381	0.023*	
C20	1.0233 (3)	−0.0197 (4)	0.52667 (19)	0.0258 (7)	
H20A	1.0682	0.0776	0.5386	0.039*	
H20B	1.0648	−0.0871	0.4920	0.039*	
H20C	1.0228	−0.0681	0.5852	0.039*	
C21	0.4537 (3)	0.4016 (4)	0.4535 (2)	0.0284 (7)	
H21A	0.4261	0.3681	0.5071	0.043*	
H21B	0.3809	0.4219	0.4021	0.043*	
H21C	0.5033	0.4950	0.4688	0.043*	
C22	1.2890 (3)	0.5444 (4)	0.9001 (2)	0.0308 (8)	
H22A	1.2676	0.6492	0.8766	0.037*	
H22B	1.3351	0.4944	0.8600	0.037*	
C23	1.3724 (3)	0.5531 (4)	0.9975 (2)	0.0258 (7)	
C24	1.3635 (3)	0.6689 (4)	1.0581 (2)	0.0262 (7)	
H24A	1.3000	0.7436	1.0396	0.031*	
C25	1.4444 (3)	0.6788 (4)	1.1448 (2)	0.0264 (7)	
H25A	1.4355	0.7599	1.1845	0.032*	
C26	1.5391 (3)	0.5715 (4)	1.1750 (2)	0.0272 (7)	
C27	1.5479 (3)	0.4538 (4)	1.1136 (2)	0.0363 (8)	
H27A	1.6114	0.3790	1.1316	0.044*	
C28	1.4656 (3)	0.4452 (4)	1.0276 (2)	0.0327 (8)	
H28A	1.4728	0.3633	0.9880	0.039*	
C29	1.6110 (3)	0.7020 (5)	1.3232 (2)	0.0388 (9)	
H29A	1.6091	0.8013	1.2929	0.058*	
H29B	1.6817	0.6985	1.3779	0.058*	
H29C	1.5335	0.6874	1.3415	0.058*	
C30	1.7049 (3)	0.4561 (5)	1.2954 (2)	0.0380 (9)	
H30A	1.6581	0.3604	1.2817	0.057*	
H30B	1.7381	0.4666	1.3621	0.057*	
H30C	1.7738	0.4553	1.2660	0.057*	
O8	0.56599 (19)	0.5632 (3)	0.73637 (15)	0.0331 (6)	
H8A	0.6175	0.5836	0.7060	0.050*	
C31	0.6124 (4)	0.6169 (5)	0.8287 (2)	0.0497 (11)	
H31A	0.6190	0.7284	0.8285	0.075*	
H31B	0.6948	0.5726	0.8556	0.075*	
H31C	0.5554	0.5863	0.8653	0.075*	

### Atomic displacement parameters (Å^2^)

**Table:**

	*U*^11^	*U*^22^	*U*^33^	*U*^12^	*U*^13^	*U*^23^
O1	0.0194 (10)	0.0258 (13)	0.0372 (12)	0.0033 (10)	0.0024 (9)	0.0088 (10)
O2	0.0221 (10)	0.0231 (13)	0.0288 (11)	−0.0015 (10)	0.0037 (8)	0.0056 (10)
O3	0.0297 (12)	0.0226 (13)	0.0381 (13)	0.0027 (10)	0.0043 (10)	0.0112 (10)
O4	0.0281 (11)	0.0225 (13)	0.0316 (12)	−0.0024 (10)	0.0011 (9)	0.0097 (10)
O5	0.0195 (10)	0.0322 (14)	0.0181 (10)	0.0066 (10)	0.0018 (8)	−0.0052 (9)
O6	0.0207 (10)	0.0286 (13)	0.0166 (9)	0.0024 (10)	0.0005 (8)	−0.0058 (9)
O7	0.0170 (9)	0.0315 (14)	0.0231 (11)	0.0070 (10)	−0.0007 (8)	−0.0064 (9)
N1	0.0167 (11)	0.0306 (16)	0.0196 (12)	−0.0074 (11)	0.0073 (9)	−0.0027 (11)
N2	0.0411 (16)	0.041 (2)	0.0359 (16)	0.0159 (16)	−0.0173 (13)	−0.0133 (15)
C1	0.0224 (14)	0.0215 (18)	0.0220 (14)	0.0001 (13)	0.0051 (12)	0.0031 (12)
C2	0.0181 (13)	0.0211 (17)	0.0172 (13)	0.0006 (13)	0.0043 (11)	−0.0007 (12)
C3	0.0172 (13)	0.0169 (16)	0.0178 (13)	0.0006 (12)	0.0064 (11)	0.0029 (12)
C4	0.0183 (13)	0.0187 (15)	0.0130 (12)	0.0001 (13)	0.0038 (10)	−0.0006 (11)
C5	0.0194 (13)	0.0197 (16)	0.0193 (13)	0.0005 (13)	0.0039 (11)	0.0014 (12)
C6	0.0242 (14)	0.0179 (16)	0.0180 (13)	−0.0013 (14)	0.0052 (11)	0.0023 (12)
C7	0.0282 (15)	0.0200 (17)	0.0272 (16)	0.0000 (14)	0.0053 (13)	0.0079 (13)
C8	0.0250 (14)	0.0194 (17)	0.0206 (14)	0.0055 (14)	0.0009 (12)	0.0031 (13)
C9	0.0197 (13)	0.0203 (17)	0.0254 (15)	0.0009 (13)	0.0031 (12)	0.0003 (12)
C10	0.0208 (13)	0.0181 (16)	0.0148 (13)	−0.0007 (12)	0.0051 (11)	−0.0010 (11)
C11	0.0157 (13)	0.0221 (17)	0.0195 (14)	−0.0003 (12)	0.0052 (11)	−0.0016 (12)
C12	0.0184 (13)	0.0203 (16)	0.0192 (13)	−0.0010 (13)	0.0045 (11)	−0.0006 (12)
C13	0.0178 (13)	0.0265 (18)	0.0218 (14)	−0.0036 (13)	0.0043 (11)	0.0017 (13)
C14	0.0153 (12)	0.0183 (16)	0.0170 (13)	−0.0022 (12)	0.0029 (10)	0.0012 (11)
C15	0.0202 (13)	0.0171 (16)	0.0193 (14)	0.0018 (13)	0.0058 (11)	0.0008 (12)
C16	0.0135 (12)	0.0205 (17)	0.0196 (14)	0.0010 (11)	0.0001 (11)	0.0023 (12)
C17	0.0170 (12)	0.0160 (15)	0.0174 (13)	−0.0026 (12)	0.0036 (11)	−0.0009 (11)
C18	0.0169 (12)	0.0178 (16)	0.0171 (13)	0.0006 (12)	0.0050 (10)	−0.0004 (11)
C19	0.0157 (12)	0.0211 (16)	0.0197 (13)	0.0028 (12)	0.0036 (10)	0.0017 (12)
C20	0.0211 (14)	0.032 (2)	0.0228 (15)	0.0063 (15)	0.0036 (12)	−0.0011 (14)
C21	0.0219 (15)	0.0275 (19)	0.0329 (17)	0.0068 (14)	0.0020 (13)	−0.0038 (15)
C22	0.0268 (15)	0.040 (2)	0.0226 (15)	−0.0134 (16)	0.0014 (12)	0.0070 (15)
C23	0.0175 (13)	0.033 (2)	0.0254 (15)	−0.0080 (14)	0.0039 (12)	−0.0035 (14)
C24	0.0191 (14)	0.0296 (19)	0.0270 (15)	0.0054 (14)	0.0008 (12)	0.0025 (14)
C25	0.0236 (14)	0.0264 (19)	0.0281 (16)	0.0018 (14)	0.0050 (12)	−0.0032 (14)
C26	0.0198 (14)	0.0272 (19)	0.0297 (16)	0.0005 (14)	−0.0022 (12)	−0.0008 (14)
C27	0.0299 (17)	0.031 (2)	0.0398 (19)	0.0139 (17)	−0.0057 (15)	−0.0049 (17)
C28	0.0289 (16)	0.028 (2)	0.0371 (18)	0.0019 (16)	0.0023 (14)	−0.0139 (16)
C29	0.0393 (19)	0.047 (2)	0.0243 (17)	0.0024 (19)	−0.0011 (14)	−0.0085 (17)
C30	0.0266 (16)	0.042 (2)	0.0385 (19)	0.0024 (17)	−0.0042 (14)	0.0112 (18)
O8	0.0234 (11)	0.0408 (15)	0.0328 (12)	−0.0059 (12)	0.0029 (9)	0.0056 (12)
C31	0.044 (2)	0.063 (3)	0.044 (2)	−0.011 (2)	0.0152 (17)	−0.014 (2)

### Geometric parameters (Å, °)

**Table:**

O1—C1	1.217 (3)	C13—H13A	0.9900
O2—C1	1.351 (3)	C13—H13B	0.9900
O2—C13	1.468 (3)	C14—C19	1.390 (4)
O3—C8	1.384 (4)	C14—C15	1.399 (4)
O3—C7	1.437 (4)	C15—C16	1.393 (4)
O4—C6	1.382 (4)	C15—H15A	0.9500
O4—C7	1.440 (4)	C16—C17	1.394 (4)
O5—C18	1.368 (3)	C17—C18	1.402 (3)
O5—C20	1.433 (3)	C18—C19	1.392 (4)
O6—C17	1.369 (3)	C19—H19A	0.9500
O6—H6A	0.8400	C20—H20A	0.9800
O7—C16	1.382 (3)	C20—H20B	0.9800
O7—C21	1.434 (4)	C20—H20C	0.9800
N1—C11	1.480 (4)	C21—H21A	0.9800
N1—C22	1.495 (4)	C21—H21B	0.9800
N1—H1A	0.8800	C21—H21C	0.9800
N2—C26	1.389 (4)	C22—C23	1.520 (4)
N2—C29	1.437 (5)	C22—H22A	0.9900
N2—C30	1.440 (5)	C22—H22B	0.9900
C1—C2	1.506 (4)	C23—C24	1.385 (5)
C2—C12	1.528 (4)	C23—C28	1.388 (5)
C2—C3	1.534 (4)	C24—C25	1.381 (4)
C2—H2A	1.0000	C24—H24A	0.9500
C3—C4	1.528 (4)	C25—C26	1.397 (4)
C3—C14	1.539 (4)	C25—H25A	0.9500
C3—H3A	1.0000	C26—C27	1.405 (5)
C4—C10	1.408 (4)	C27—C28	1.379 (4)
C4—C5	1.413 (4)	C27—H27A	0.9500
C5—C6	1.361 (4)	C28—H28A	0.9500
C5—H5A	0.9500	C29—H29A	0.9800
C6—C8	1.392 (4)	C29—H29B	0.9800
C7—H7A	0.9900	C29—H29C	0.9800
C7—H7B	0.9900	C30—H30A	0.9800
C8—C9	1.355 (4)	C30—H30B	0.9800
C9—C10	1.422 (4)	C30—H30C	0.9800
C9—H9A	0.9500	O8—C31	1.431 (4)
C10—C11	1.528 (4)	O8—H8A	0.8400
C11—C12	1.522 (4)	C31—H31A	0.9800
C11—H11A	1.0000	C31—H31B	0.9800
C12—C13	1.518 (4)	C31—H31C	0.9800
C12—H12A	1.0000		
			
C1—O2—C13	109.3 (2)	C15—C14—C3	119.7 (2)
C8—O3—C7	104.5 (2)	C16—C15—C14	119.5 (3)
C6—O4—C7	104.6 (2)	C16—C15—H15A	120.2
C18—O5—C20	116.2 (2)	C14—C15—H15A	120.2
C17—O6—H6A	109.5	O7—C16—C15	125.0 (3)
C16—O7—C21	116.9 (2)	O7—C16—C17	113.6 (2)
C11—N1—C22	113.6 (2)	C15—C16—C17	121.4 (2)
C11—N1—H1A	123.2	O6—C17—C16	123.3 (2)
C22—N1—H1A	123.2	O6—C17—C18	118.0 (2)
C26—N2—C29	120.1 (3)	C16—C17—C18	118.7 (2)
C26—N2—C30	119.5 (3)	O5—C18—C19	124.1 (2)
C29—N2—C30	118.8 (3)	O5—C18—C17	116.0 (2)
O1—C1—O2	120.5 (3)	C19—C18—C17	119.8 (3)
O1—C1—C2	129.7 (3)	C14—C19—C18	121.2 (2)
O2—C1—C2	109.7 (2)	C14—C19—H19A	119.4
C1—C2—C12	102.3 (2)	C18—C19—H19A	119.4
C1—C2—C3	120.4 (2)	O5—C20—H20A	109.5
C12—C2—C3	112.5 (3)	O5—C20—H20B	109.5
C1—C2—H2A	106.9	H20A—C20—H20B	109.5
C12—C2—H2A	106.9	O5—C20—H20C	109.5
C3—C2—H2A	106.9	H20A—C20—H20C	109.5
C4—C3—C2	107.5 (2)	H20B—C20—H20C	109.5
C4—C3—C14	110.6 (2)	O7—C21—H21A	109.5
C2—C3—C14	115.2 (2)	O7—C21—H21B	109.5
C4—C3—H3A	107.8	H21A—C21—H21B	109.5
C2—C3—H3A	107.8	O7—C21—H21C	109.5
C14—C3—H3A	107.8	H21A—C21—H21C	109.5
C10—C4—C5	120.4 (3)	H21B—C21—H21C	109.5
C10—C4—C3	122.6 (3)	N1—C22—C23	112.2 (2)
C5—C4—C3	116.8 (2)	N1—C22—H22A	109.2
C6—C5—C4	118.3 (3)	C23—C22—H22A	109.2
C6—C5—H5A	120.8	N1—C22—H22B	109.2
C4—C5—H5A	120.8	C23—C22—H22B	109.2
C5—C6—O4	129.3 (3)	H22A—C22—H22B	107.9
C5—C6—C8	121.2 (3)	C24—C23—C28	117.3 (3)
O4—C6—C8	109.5 (3)	C24—C23—C22	122.8 (3)
O3—C7—O4	107.4 (2)	C28—C23—C22	119.8 (3)
O3—C7—H7A	110.2	C25—C24—C23	121.8 (3)
O4—C7—H7A	110.2	C25—C24—H24A	119.1
O3—C7—H7B	110.2	C23—C24—H24A	119.1
O4—C7—H7B	110.2	C24—C25—C26	121.1 (3)
H7A—C7—H7B	108.5	C24—C25—H25A	119.5
C9—C8—O3	127.7 (3)	C26—C25—H25A	119.5
C9—C8—C6	122.6 (3)	N2—C26—C25	121.7 (3)
O3—C8—C6	109.7 (3)	N2—C26—C27	121.2 (3)
C8—C9—C10	117.9 (3)	C25—C26—C27	117.1 (3)
C8—C9—H9A	121.0	C28—C27—C26	121.0 (3)
C10—C9—H9A	121.0	C28—C27—H27A	119.5
C4—C10—C9	119.6 (3)	C26—C27—H27A	119.5
C4—C10—C11	124.3 (3)	C27—C28—C23	121.8 (3)
C9—C10—C11	116.1 (2)	C27—C28—H28A	119.1
N1—C11—C12	114.0 (3)	C23—C28—H28A	119.1
N1—C11—C10	109.1 (2)	N2—C29—H29A	109.5
C12—C11—C10	109.6 (2)	N2—C29—H29B	109.5
N1—C11—H11A	108.0	H29A—C29—H29B	109.5
C12—C11—H11A	108.0	N2—C29—H29C	109.5
C10—C11—H11A	108.0	H29A—C29—H29C	109.5
C13—C12—C11	120.2 (2)	H29B—C29—H29C	109.5
C13—C12—C2	100.8 (2)	N2—C30—H30A	109.5
C11—C12—C2	111.4 (2)	N2—C30—H30B	109.5
C13—C12—H12A	107.9	H30A—C30—H30B	109.5
C11—C12—H12A	107.9	N2—C30—H30C	109.5
C2—C12—H12A	107.9	H30A—C30—H30C	109.5
O2—C13—C12	103.8 (2)	H30B—C30—H30C	109.5
O2—C13—H13A	111.0	C31—O8—H8A	109.5
C12—C13—H13A	111.0	O8—C31—H31A	109.5
O2—C13—H13B	111.0	O8—C31—H31B	109.5
C12—C13—H13B	111.0	H31A—C31—H31B	109.5
H13A—C13—H13B	109.0	O8—C31—H31C	109.5
C19—C14—C15	119.3 (3)	H31A—C31—H31C	109.5
C19—C14—C3	120.9 (2)	H31B—C31—H31C	109.5
			
C13—O2—C1—O1	175.0 (3)	C3—C2—C12—C13	164.5 (2)
C13—O2—C1—C2	−2.7 (3)	C1—C2—C12—C11	162.5 (2)
O1—C1—C2—C12	162.1 (3)	C3—C2—C12—C11	−66.8 (3)
O2—C1—C2—C12	−20.5 (3)	C1—O2—C13—C12	25.1 (3)
O1—C1—C2—C3	36.4 (5)	C11—C12—C13—O2	−158.8 (2)
O2—C1—C2—C3	−146.1 (2)	C2—C12—C13—O2	−36.0 (3)
C1—C2—C3—C4	172.5 (2)	C4—C3—C14—C19	−27.4 (4)
C12—C2—C3—C4	51.8 (3)	C2—C3—C14—C19	94.8 (3)
C1—C2—C3—C14	48.7 (3)	C4—C3—C14—C15	149.1 (3)
C12—C2—C3—C14	−72.0 (3)	C2—C3—C14—C15	−88.8 (3)
C2—C3—C4—C10	−22.5 (3)	C19—C14—C15—C16	−0.1 (4)
C14—C3—C4—C10	104.1 (3)	C3—C14—C15—C16	−176.7 (3)
C2—C3—C4—C5	162.2 (2)	C21—O7—C16—C15	7.7 (4)
C14—C3—C4—C5	−71.3 (3)	C21—O7—C16—C17	−173.9 (2)
C10—C4—C5—C6	0.7 (4)	C14—C15—C16—O7	179.2 (3)
C3—C4—C5—C6	176.2 (2)	C14—C15—C16—C17	0.9 (4)
C4—C5—C6—O4	−177.2 (3)	O7—C16—C17—O6	0.2 (4)
C4—C5—C6—C8	−1.3 (4)	C15—C16—C17—O6	178.6 (3)
C7—O4—C6—C5	−171.1 (3)	O7—C16—C17—C18	179.9 (3)
C7—O4—C6—C8	12.7 (3)	C15—C16—C17—C18	−1.7 (4)
C8—O3—C7—O4	20.3 (3)	C20—O5—C18—C19	−7.7 (4)
C6—O4—C7—O3	−20.4 (3)	C20—O5—C18—C17	172.4 (3)
C7—O3—C8—C9	170.1 (3)	O6—C17—C18—O5	1.3 (4)
C7—O3—C8—C6	−12.6 (3)	C16—C17—C18—O5	−178.5 (2)
C5—C6—C8—C9	0.8 (5)	O6—C17—C18—C19	−178.6 (3)
O4—C6—C8—C9	177.4 (3)	C16—C17—C18—C19	1.7 (4)
C5—C6—C8—O3	−176.6 (3)	C15—C14—C19—C18	0.1 (4)
O4—C6—C8—O3	−0.1 (3)	C3—C14—C19—C18	176.6 (3)
O3—C8—C9—C10	177.3 (3)	O5—C18—C19—C14	179.2 (3)
C6—C8—C9—C10	0.4 (5)	C17—C18—C19—C14	−0.9 (4)
C5—C4—C10—C9	0.4 (4)	C11—N1—C22—C23	−148.1 (3)
C3—C4—C10—C9	−174.8 (3)	N1—C22—C23—C24	−89.1 (4)
C5—C4—C10—C11	−179.2 (3)	N1—C22—C23—C28	93.5 (4)
C3—C4—C10—C11	5.6 (4)	C28—C23—C24—C25	0.7 (5)
C8—C9—C10—C4	−0.9 (4)	C22—C23—C24—C25	−176.7 (3)
C8—C9—C10—C11	178.7 (2)	C23—C24—C25—C26	0.0 (5)
C22—N1—C11—C12	−86.8 (3)	C29—N2—C26—C25	3.5 (5)
C22—N1—C11—C10	150.3 (3)	C30—N2—C26—C25	168.9 (3)
C4—C10—C11—N1	109.5 (3)	C29—N2—C26—C27	−178.5 (3)
C9—C10—C11—N1	−70.1 (3)	C30—N2—C26—C27	−13.1 (5)
C4—C10—C11—C12	−16.0 (4)	C24—C25—C26—N2	177.9 (3)
C9—C10—C11—C12	164.4 (2)	C24—C25—C26—C27	−0.2 (5)
N1—C11—C12—C13	39.2 (3)	N2—C26—C27—C28	−178.4 (4)
C10—C11—C12—C13	161.8 (2)	C25—C26—C27—C28	−0.3 (5)
N1—C11—C12—C2	−78.3 (3)	C26—C27—C28—C23	1.1 (6)
C10—C11—C12—C2	44.3 (3)	C24—C23—C28—C27	−1.2 (5)
C1—C2—C12—C13	33.8 (3)	C22—C23—C28—C27	176.2 (3)

### Hydrogen-bond geometry (Å, °)

**Table:**

*D*—H···*A*	*D*—H	H···*A*	*D*···*A*	*D*—H···*A*
O8—H8A···O1	0.84	1.95	2.777 (3)	170.
O6—H6A···O8^i^	0.84	1.97	2.756 (3)	156.
C7—H7B···N1^ii^	0.99	2.62	3.473 (4)	145.
C13—H13B···O6^iii^	0.99	2.52	3.463 (4)	159.
C24—H24A···O3^iv^	0.95	2.35	3.242 (4)	156.

Symmetry codes: (i) −*x*+1, *y*−1/2, −*z*+1; (ii) *x*, *y*−1, *z*; (iii) −*x*+2, *y*+1/2, −*z*+1; (iv) *x*, *y*+1, *z*.
